# Book Reviews

**Published:** 1896-07

**Authors:** 


Medical Jurisprudence, Forensic Medicine and Toxicology. By R. A. Witthaus, A. M., M. D., and Tracy C. Becker, A. B., LL. B., and a staff of collaborators. In four royal 8vo. volumes. Volume III. Forensic Medicine (continued). New York: William Wood & Co. 1896.

The third volume of this important work opens with a section on the medico-legal relations of vision and audition, and of injuries to the eye and ear, written by J. H. Woodward, M. D., professor of ophthalmology and laryngology in the University of Vermont. Professor Woodward devotes eight chapters, comprising 128 pages, to the consideration of his subject, thus making it a full and well-nigh complete exposition of a hitherto little understood branch of legal medicine. It will prove not only interesting to the medical jurist, but also to the ophthalmologist and otologist.

The next subject considered is the medico-legal aspect of insurance. This section comprises forty pages and is a product of the joint authorship of David Murray and Guy J. Edwords, of the New York bar. In dealing with the question of residence, the authors cite the following case of interest: When Boss Tweed made his escape from custody in September, 1875, he fled to Cuba and thence to Spain, where he was captured at Vigo. A suit on a policy of insurance taken out by him in 1868 was successfully resisted on the ground that he had violated the condition in the policy that he should not "without the written consent of this company, previously obtained, travel upon the high seas, except between coastwise parts of the United States."

We now come to a consideration of the medical aspects of insanity in its relations to medical jurisprudence, written by Edward D. Fisher, professor of nervous and mental diseases, University of the City of New York. Always an interesting writer, Dr. Fisher was never more so than in the 200 pages set apart to the consideration of this topic, perhaps the most difficult question in medical jurisprudence. Dr. Fisher's classifications are not only scientific but concise, while his definitions of the several subdivisions of insanity are at once learned and terse. This section will prove helpful in a high degree to every medical witness called to testify in regard to insanity.

Following the preceding in its natural order of sequence we find mental unsoundness in its legal relations treated of by Tracy C. Becker, one of the editors. Mr. Becker, who is accustomed to teaching, is a writer of great clearness and has done himself special credit in the 170 pages here presented to his readers. Testamentary law, so intimately related to this subject, is considered with some detail, while all through the section references to interesting illustrative cases are numerous. Both lawyers and doctors will find instructive research in this section.

Finally, we note that the care and custody of incompetent persons and their estates is dealt with by Mr. Goodwin Brown, of the New York state lunacy commission, in a section of 145 pages. In this we find a digest of the statutes of all the states relating to the subject matter of this section, compiled by Frank D. Gilbert, of the New York statutory revision commission, which will prove of special interest to lawyers. The volume closes with a table of cases cited and an ample index. It is a fitting complement to those that have preceded it and is a continuation of a superb and comprehensive treatise.

———

TWENTIETH CENTURY PRACTICE. An International Encyclopedia of Modern Medical Science. By Leading Authorities of Europe and America. Edited by Thomas L. Stedman, M. D., New York City. Twenty volumes. Volume V. Diseases of the Skin. New York : William Wood & Co. 1896.

As has already been explained in these pages, the publishers found it necessary to issue Volume VI. out of its regular order, hence it appeared and was noticed previous to the present number.

The volume under consideration is devoted entirely to diseases of the skin and is the largest single volume set apart to this subject that has yet appeared. The first section treats of the anatomy of the skin and its appendages, and is prepared by Charles W. Allen, of New York. The author gives a succinct account of the subject in well-chosen terms.

The next subject treated is parasitic diseases and is written by L. Duncan Bulkley, also of New York, who by heredity and education is a dermatologist par excellence and an author with facile pen and fluent thought. A knowledge of parasitic diseases is alike necessary to the general physician and the dermatologist ; both will find them here discussed in their latest light.

We next come to a section on erythematous affections, written by Henry H. Whitehouse, also of New York. This is followed by a section on eczema and dermatitis by James Nevins Hyde, of Chicago. A knowledge of the multifarious manifestations of the quite common disease known as eczema is essential for the practitioner of general medicine and he should study its history, manifestations and treatment as set forth by Hyde with assiduity.

Next follow in consecutive order sections devoted to squamous affections by H. Radcliffe Crocker, of London ; papular affections by L. Brocq, of Paris ; bullous affections by Henry H. Whitehouse, of New York ; pustular affections by the same author ; phlegmonous and ulcerative affections by Crocker ; diseases of the sebaceous glands and diseases of the sweat glands by Arthur Van Harlingen, of Philadelphia ; diseases of the hair and nails (this latter of great importance and well handled) by Douglass W. Montgomery, of San Francisco. Then follows an important section in which benign neoplasms are considered in much detail by John T. Bowen, of Boston. Moris Kaposi, of Vienna, contributes a short but well-illustrated section devoted to the consideration of xeroderma pigmentosum.

Finally, the dermatoneuroses are taken up by H. Leloir, of Lille, the consideration of which continues to the end of the volume. Such a treatise on dermatology is a marvel in design and execution and challenges admiration for authors, editor, and publishers.

---

New York State Commission in Lunacy. Sixth Annual Report, October 1, 1893, to September 30, 1894. T. E. McGarr, secretary. Transmitted to the Legislature May 24, 1895. Albany: James B. Lyon, State Printer. 1895.

This excellent report sets forth the workings of the state hospitals for the insane for the period indicated in the title. It merits the careful examination of every physician or other person interested in or engaged in the care of lunatics. The economics of the system, as applied to the state of New York, are well digested and considered in detail. It is the most complete report on the subject that has yet been issued.

---

International Clinics. A Quarterly of Clinical Lectures on Medicine, Neurology, Surgery, Gynecology, Obstetrics, Ophthalmology, Laryngology, Pharyngology, Rhinology, Otology and Dermatology, and specially prepared articles on treatment. By professors and lecturers in the leading medical colleges of the United States, Germany, Austria, France, Great Britain and Canada. Edited by Judson Daland, M. D., (Univ. of Penna.) Philadelphia, Instructor in Clinical Medicine and Lecturer on Physical Diagnosis in the University of Pennsylvania; Assistant Physician to the Hospital of the University of Pennsylvania; Director of the Stetson Laboratory of Hygiene; Fellow of the College of Physicians of Philadelphia. J. Mitchell Bruce, M. D., F. R. C. P., London, England, Physician to, and Lecturer on, the Principles and Practice of Medicine at the Charing Cross Hospital. David W. Finlay, M. D., F. R. C. P., Aberdeen, Scotland, Professor of Practice of Medicine in the University of Aberdeen; Physician to, and Lecturer on, Clinical Medicine in the Aberdeen Royal Infirmary; Consulting Physician to the Royal Hospital for Diseases of the Chest, London. Volume I. Sixth series. 1896. Royal 8vo, pp. xii.—344. Philadelphia: J. B. Lippincott Co. 1896.

In the first volume of the sixth series we find a continuation of the same system that has pervaded these clinics since their introduction to the profession several years ago, with the addition of specially prepared articles on treatment. These latter will prove of interest to younger physicians. The Buffalo contingent contributing to this volume is composed of Drs. Benedict, Mann, Park and Stockton. We find also the following prominent names among the contributors: Drs. Clinton Cushing, San Francisco; H. H. Grant and Joseph M. Mathews, Louisville; Prof. Potain, Paris; Dr. John B. Roberts, Philadelphia; Granger Stewart, Edinburg; Prof. Vulliet, Geneva (now deceased), and Dr. James T. Whittaker, Cincinnati. There are a number of others, but we have not space to mention all.

This volume is quite well illustrated and will easily find its way to the bookshelves of progressive physicians.

Proceedings of the Orleans Parish Medical Society for 1894. Augustus McShane, M. D., secretary. Vol. II. New Orleans. L. Graham & Son, Printers. 1895.

This volume contains the reports of the routine business of the society, together with reports and discussions of several interesting scientific articles.

ATLAS OF TRAUMATIC FRACTURES AND DISLOCATIONS. With a Brief Treatise. By H. HELFERICH, M. D., Professor at the University of Greifswald. With 166 illustrations, after original drawings by Dr. Joseph Trumpp. New York: William Wood & Co. 1896.

Fractures and dislocations furnish the Scylla and Charybdis of the young practitioner of medicine. In this book both he and the student entering upon this important field of surgery will find an opportunity to study the subjects with that anatomical detail that is so necessary to their clear understanding. The drawings for the most part are original, made by the author from recent specimens and demonstrate the several branches of the subject with unusual clearness. The aid of such a work to the student and to the graduate just entering upon the practice of surgery is beyond calculation and stands nowise in relation to its cost price. It does not take the place of clinical studies, nor of such a classic treatise as Hamilton's, but it is a most useful adjunct to those two indispensable methods of acquiring knowledge on the subject of fractures and dislocations.

PROCEEDINGS of the Twenty-first and Twenty-second Annual Meetings of the Oregon State Medical Society, held at Portland, May 31, June 1, 2, 1894, and June 11 and 12, 1895. Volume XX. Edited by E. F. Tucker, M. D., secretary, Portland. 1895.

The book contains full reports of the papers read at the annual meetings of '94 and '95, together with discussions of the same. It is an interesting collection of medical essays, covering a considerable range of topics.

Transactions of the State Medical Society of Wisconsin for the Year 1895. Volume XXIX. Constitution and by-laws and list of members. Charles S. Sheldon, M. D., secretary. Madison, Wisconsin: Tracy, Gibbs & Co., Printers and Stereotypers. 1895.

This record of the forty-ninth meeting of this society is most satisfactory. It contains fifty-seven papers, many of which were interestingly and instructively discussed. The secretary has performed his work in a thorough-going manner. No medical society can prosper unless its secretary has executive capacity and literary ability. Both of these qualifications are demonstrated in the book before us. As is the case with most state medical societies, however, the number of members in attendance at this meeting was comparatively small. The medical profession very generally seems apathetic in regard to its state societies, a condition we hope to see improved as the years advance. The state medical society influences the medical status of the profession and in a large measure controls medical legislation; hence it should enroll upon its membership list a large proportion of its resident medical population.

TRANSACTIONS OF THE COLORADO STATE MEDICAL SOCIETY. Twenty-fifth annual convention, held in Denver, Col., June 18, 19, 20, 1895. By-laws and list of members. Edwin R. Axtell, M. D., secretary. Denver, Col. : Published for the Society.

This handsome volume indicates the progressiveness of the medical profession of this growing commonwealth. The papers are excellent in form and embrace almost every kind of disease in medicine and surgery, and some of the discussions are also spirited and able. A unique feature of this book consists in publishing half-toned portraits of the twenty-five ex-presidents of the society, six of whom are deceased. The value of this portrait gallery will become inestimable in after years. The book is handsomely printed and well bound.

PROCEEDINGS OF THE TWENTY-SECOND SESSION OF THE FLORIDA MEDICAL ASSOCIATION, held at Gainesville, Fla., April 16, 17 and 18, 1895. Edited by J. D. FERNANDEZ, M. D., Secretary.

This association in the publication of this brochure of 128 pages demonstrates its activity and scientific *esprit de corps.* In addition to the proceedings, that occupy thirty-four pages of the pamphlet, eighteen medical papers are published, dealing with almost every department of medicine. It is a creditable showing for the Floral state.

---

DIETS FOR INFANTS AND CHILDREN IN HEALTH AND DISEASE. By LOUIS STARR, M. D., Editor American Text-book of the Diseases of Children. Price, $1.25 net. Philadelphia : W. B. Saunders, 925 Walnut street. 1896.

This little book is made up of detachable leaves, on which are printed the diet lists for children, sick and well, ranging in age from birth to late childhood. On the leaves prepared for younger children the ingredients only are given, the quantities of each being left for the physician to determine. Space is also left for hygienic directions. Detachable recipes for various broths, and the like, are also included in the volume, which should prove very helpful, insuring as its use would an accurate understanding of the physician's orders concerning the child's feeding and general management.

TRANSACTIONS of the Texas State Medical Association. Twenty-seventh annual session, held at Dallas, Texas, April 23, 24, 25, 26, 1895. H. A. West, M. D., secretary. Galveston, Texas: Knapp Bros., Printers and Publishers. 1895.

The book contains the list of officers for the year 1895-6, the constitution and by-laws of the association and minutes of the twenty-seventh annual meeting. The papers read at this session are reported in full. A picture of Dr. P. C. Coleman, the president, faces the title page.

